# Test-retest reliability of resting-state functional magnetic resonance imaging during deep brain stimulation for Parkinson’s disease

**DOI:** 10.1016/j.nicl.2026.103973

**Published:** 2026-02-18

**Authors:** Skyler Deutsch, Juhi Mehta, Lee B. Reid, Andrea Fuentes, Sarah Wang, John Kornak, Philip A. Starr, Jill L. Ostrem, Doris D. Wang, Ian O. Bledsoe, Melanie A. Morrison

**Affiliations:** a Department of Radiology & Biomedical Imaging, University of California San Francisco, San Francisco, USA; b Department of Neurology & Neurological Sciences, Stanford University, Stanford, CA, USA; c Department of Neurology, University of California San Francisco, San Francisco, CA, USA; d Department of Epidemiology and Biostatistics, University of California San Francisco, San Francisco, CA, USA; e Department of Neurological Surgery, University of California San Francisco, San Francisco, CA, USA

**Keywords:** Functional magnetic resonance imaging, Test-retest reliability, Deep brain stimulation, Parkinson's disease, Metal artifact, Open-source

## Abstract

**Background:**

Patients implanted with modern deep brain stimulation (DBS) hardware can now undergo functional magnetic resonance imaging (fMRI), leading to its increased used to study DBS’ mechanisms and predict optimal therapy settings. To accurately interpret fMRI data and realize its clinical potential for DBS, a better understanding of reliability is needed.

**Methods:**

Sixteen patients with Parkinson’s disease (PD) and DBS targeting the subthalamic nucleus or pallidum underwent 3T test–retest resting-state fMRI with and without concurrent stimulation. Effects of stimulation and device-metal artifacts on reliability of fMRI brain connectivity and moment-to-moment brain variability were explored, plus factors influencing between-subject variations in reliability such as motion.

**Results:**

The brain variability fMRI metric yielded higher intra-class correlation coefficients than the connectivity metric (range across whole brain, motor, limbic, and cognitive networks: 0.36–0.85 and 0.68–0.99, respectively). Average network connectivity appeared less reproducible when DBS was ON versus OFF during fMRI, and fMRI metric reliability for brain areas affected by metal artifacts was significantly higher (brain variability) or lower (connectivity) than unaffected areas (p_uncorrected_ < 0.05). Motion and DBS target best explained between-subject variations.

**Conclusion:**

DBS hardware and active stimulation may alter fMRI reliability**.** To develop clinically useful fMRI biomarkers for DBS and aid assessments of reproducibility across studies, the reliability of single study results need reporting.

## Highlights

• fMRI metric reproducibility may differ with DBS turned on versus off.
• DBS metal alters fMRI signal and may impact reliability of fMRI metrics.
• Head motion, the DBS target, and total response to DBS may influence fMRI reliability.

## 1 Introduction

Functional magnetic resonance imaging (fMRI) continues to be leveraged as a discovery tool to decode brain-behavior interactions, (Gordon et al., 2023) measure functional alterations associated with medical conditions, (Xia et al., 2019) and evaluate therapeutic mechanisms for predicting patient outcomes (Reggente et al., 2018). Both resting-state and block-design fMRI paradigms are used to investigate the therapeutic mechanisms of deep brain stimulation (DBS), a surgical neuromodulation therapy, and explain associated variance in patient outcomes (Loh et al., 2022). Advances in DBS hardware now permit patients with specific devices to undergo 1.5T and 3T MRI (Paff et al., 2020); studies with concurrent stimulation and fMRI have reinforced the idea that wide-spread alteration of targeted network oscillatory activity underlies symptom improvement with DBS (Herrington et al., 2016, Horn and Fox, 2020). Clinical use cases have emerged, too, with several studies demonstrating the potential for fMRI to streamline traditional trial-and-error-based DBS programming strategies (Boutet et al., 2021, DiMarzio et al., 2020). For example, Boutet et al showed that clinically optimal versus non-optimal stimulation settings (i.e., electrode contacts and voltages) can be predicted for the individual patient with good accuracy using supervised learning models trained on DBS-fMRI response patterns (Boutet et al., 2021). As clinical use of DBS continues to grow, adoption of these much-needed streamlined approaches will depend on their reliability, which in turn depends on the reliability of functional brain response to DBS measured by fMRI (that is, the consistency of fMRI responses across repeated measurements). Having shaped our current understanding of DBS therapeutic mechanisms, these fMRI measures and their reliability are also relevant to the development of robust prognostic markers for DBS candidate selection.

Brain alterations associated with healthy aging and medical conditions can directly and indirectly reduce fMRI reliability. A brain tumor, for example, might alter one’s ability to follow instructions and disrupt the neurovascular coupling that fMRI depends upon (Morrison et al., 2016). Motor circuit dysfunction in Parkinson’s disease (PD) is another example where functional heterogeneity and uncontrollable movement can threaten fMRI reliability and contribute to non-reproducible findings across datasets (Badea et al., 2017). In populations like PD receiving DBS, the implanted hardware cause magnetic susceptibility artifacts that can impact fMRI reliability even further. Acquisition and preprocessing strategies have been proposed to mitigate these metal artifacts that present as localized signal loss and geometric warping of images and become more pronounced at higher MRI field strengths (In et al., 2017, Lehto et al., 2017). Some groups performing connectivity analyses have alternatively opted to use coarse parcellation schemes that average out signal from voxels containing metal artifacts, if not excluding the affected voxels altogether (Mueller et al., 2018, Horn et al., 2019, Zhang et al., 2021). In addition to the presence of metal in the brain, when DBS is turned on, it exerts time-varying modulatory effects on neural activity (Sinclair et al., 2019, Wiest et al., 2020). This time-varying effect and any accompanying changes in motor symptoms may independently reduce or contribute noise to the fMRI signal. Since functional brain response to DBS is typically inferred from changes in the fMRI signal (or a derived fMRI metric) when DBS is turned on versus off, investigating potential differences in fMRI reliability across these stimulation conditions is essential and could redefine how we use and interpret fMRI findings to advance DBS.

To better understand data reliability in DBS imaging research, here we conducted an opportunistic resting-state fMRI (rsfMRI) test–retest study in 16 patients with PD receiving DBS targeting the subthalamic nucleus (STN) or globus pallidus internus (GPi). We first evaluated the effects of stimulation and DBS metal artifact on the reliability of fMRI measures of connectivity and brain variability, before exploring factors that might explain between-subject variations in fMRI reliability. Connectivity measures have been commonly used to detect network reorganization in response to stimulation, (Loh et al., 2022) and thus establishing their reliability across different stimulation conditions is crucial for interpreting findings. Brain variability, which measures moment-to-moment fluctuations in fMRI signal, is an additional promising metric thought to reflect the underlying dynamic range of neural activity and, thus, the capacity for neural adaptation (Månsson et al., 2022, Garrett et al., 2013). Recent studies have demonstrated that brain variability critically facilitates network organization and integration, (Baracchini et al., 2021, Garrett et al., 2018) and helps predict and evaluate treatment outcomes (Månsson et al., 2022, Morrison et al., 2022).

## 2 Methods

### 2.1 Participants and study design

Following IRB approval, 16 patients with PD (median age: 66.4 years, range: 50.3–76.4 years, three females) implanted with 3T MRI-conditional Medtronic Percept PC neurostimulators and Activa or SenSight leads, consented to participate in this study at the University of California, San Francisco between November 2022 and June 2023. All patients met institutional DBS inclusion criteria (e.g., stable cognition, motor symptom responsiveness to levodopa) and had no other contraindications to MRI. As presented in Table 1, patients’ leads had been implanted either in the STN (one unilateral, six bilateral) or GPi (one unilateral, eight bilateral). All patients received continuous stimulation as part of clinical care for at least four months (median: 15.3 months, range: 4.3–65.5 months), except for one enrolled in an adaptive DBS trial. As part of this study, each patient underwent a neurological assessment followed by structural MRI then rsfMRI twice with DBS turned off and twice with DBS turned on. Three patients returned 2.3–6.4 months later for serial imaging and neurological assessment, opportunistically enabling exploration of data reliability over a longer interval.

**Table 1:** Cross-sectional patient characteristics.

Characteristic^a^	Subject Proportions
	N = 16
**Age**, mean (range)	64.9 (50.3–76.4)
**Female**, count	3
**Race**, count	
White	15
Black	1
**Target**, count STN GPi	7 9
**Lead configuration**, count Bilateral Unilateral	15 1
**LEDD (mg)**, mean total (range)	774 (260–1589)
**MDS-UPDRS improvement score (%)**, mean total (range)	
part III acute^b^	38.2 (23.5–57.1)
**MDS-UPDRS raw score**, mean (range)	
*DBS-OFF*	
part III total	46 (27–63)
part III bradykinesia	25 (14–33)
part III rigidity	9 (3–13)
part III tremor	8 (3–19)
part III axial	5 (1–9)
*DBS-ON*	
part III total	29 (15–42)
part III bradykinesia	17 (8–26)
part III rigidity	5 (1–10)
part III tremor	4 (0–11)
part III axial	4 (1–7)

a Characteristics are based on the time of the primary MRI. No serial data are included.

b DBS-OFF vs. DBS-ON.

### 2.2 MRI protocol

Our workflow for imaging patients with DBS is available on GitHub along with materials needed to replicate our methods: https://github.com/Radiology-Morrison-lab-UCSF/DBS_fMRI_Reproducibility. The raw fMRI data from consented patients can be accessed via OpenNeuro Accession Number: ds005906.

**Fig. 1:**
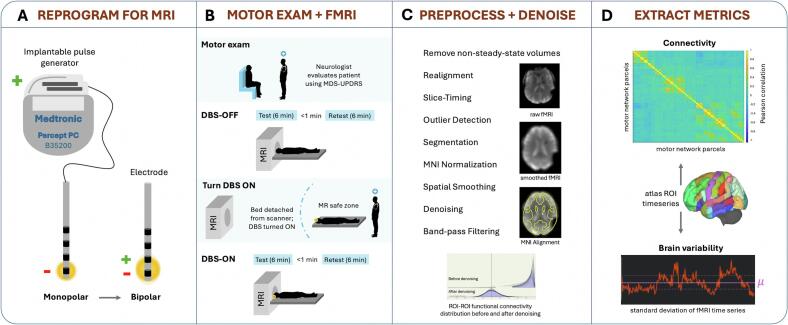
Study workflow and fMRI methods. A. Patients’ DBS settings were reprogrammed from a clinical monopolar configuration to an MRI-safe bipolar configuration. B. A motor exam evaluated symptom response to DBS before test and retest fMRI data were acquired with DBS turned off, then on. C–D. fMRI time series data were preprocessed and denoised using the CONN toolbox, and connectivity and brain variability metrics were extracted for reproducibility analysis.

To maximize comfort and minimize the potential for motion during scanning, patients were examined while on their routine PD medications (Table 1). Following MRI screening, a study neurologist tested each patient’s device for disrupted electrical connections (open circuits) and programmed them into an MRI-safe bipolar configuration (where both the anode and cathode are electrode contacts on the DBS lead), providing similar motor symptom relief to patients’ clinical monopolar settings (where the cathode is an electrode contact on the lead and the anode is the implanted neurostimulator case; Fig. 1A). This was an iterative personalized process whereby symptom relief was evaluated in real-time using the motor portion of the Movement Disorder Society-Unified PD Rating Scale (MDS-UPDRS-III) (Goetz et al., 2008). After reprogramming and neurological assessment (see 2.3.), the device was kept off to allow for washout of DBS effects during the initial structural T1-weighted (T1w) sequence (subject washout range was 5–63 min due to scanner availability and downtime), but to also optimize patient comfort, minimize heating risks, and ensure complete data acquisition within scanner time constraints. Using a 3T General Electric Discovery MR750 whole-body scanner in first-level controlled mode (max B1 + rms < 2μT, max spatial field gradient = 20 T/m), equipped with a whole-body transmit coil and a 32-channel Nova Medical receive-only head-coil, a 3.5-minute T1w gradient echo sequence was first acquired (inversion time (TI)/repetition time (TR)/echo time (TE) = 600/8.56/3.36 ms, voxel size = 1x1x1.2 mm, field of view (FOV) = 256x256mm^2^, flip angle (FA) = 12°, ASSET in-plane acceleration = 2). With DBS still turned off, two 6-minute T2*w gradient-echo echo-planar rsfMRI sequences were acquired < 1 min apart (Fig. 1B**;** TR/TE = 2150/29 ms, voxel size = 3.75x3.75x4mm, FOV = 240x240mm^2^, FA = 84°, ARC in-plane acceleration = 2). The patient was then removed from the scanner to turn the DBS device on and the rsfMRI sequences were repeated, resulting in 27.5 min of total scanning time, plus additional time for brain (re-)localization and calibration sequences. Per safety guidelines, the protocol did not exceed 30 min of active scan time. Under this time constraint, we prioritized dense fMRI sampling and therefore did not collect field maps for distortion correction. Supporting this decision is prior evidence showing that standard corrective methods (and even advanced acquisition and postprocessing strategies) cannot adequately compensate for the predominate signal-loss artifacts introduced by DBS hardware (In et al., 2017, Lehto et al., 2017). This underscores the importance of characterizing the reliability and potential utility of this unavoidable, artifact-prone fMRI signal at the site of DBS hardware.

### 2.3 Neurological assessment

Immediately before imaging, a study neurologist administered the MDS-UPDRS-III exam while DBS was turned off, then on in the MR-safe bipolar configuration. Exam scores were used to calculate patients’ acute (DBS-OFF_ON-MEDS_ vs. DBS-ON_ON-MEDS_) motor symptom improvement with DBS as: improvement=((MDS-UPDRSIII_OFF_–MDS-UPDRSIII_ON_)/MDS-UPDRSIII_OFF_)x100. Raw MDS-UPDRS-III symptom subscores were also used as a proxy of disease severity in our variance analysis (see 2.5.3.) and calculated as in Li et al (see **Supplementary**) (Li et al., 2018).

### 2.4 fMRI metrics for reliability testing

#### 2.4.1 Preprocessing

Functional and T1w data were preprocessed using the default preprocessing pipeline for volume-based analyses in CONN (v22a), (Whitfield-Gabrieli and Nieto-Castanon, 2012) an open-source SPM-based (v12) (Friston et al., 1994) toolbox. The only addition to this pipeline was the initial removal of two non-steady-state fMRI volumes (Fig. 1C**;** see **Supplementary** for full preprocessing details). For this study, we manually imported the Zhang atlas (Zhang et al., 2018) into CONN, derived from the automated anatomical labeling atlas (Rolls et al., 2020) and iron-sensitive MRI for accurate segmentation of deep grey matter structures.

After preprocessing, the CONN toolbox was used to denoise the smoothed fMRI data. The goal was to optimize the removal of noise components and center positively-skewed distributions of pairwise fMRI correlations (Fig. 1C) while maintaining > 30 degrees of freedom. Linear regression removed confounders related to scanner hardware, physiology, and/or motion before bandpass filtering at 0.01–0.1 Hz to isolate hemodynamic signal of interest. For exploratory purposes, we also processed the data using a wider filter of 0.01–0.25 Hz. Specific confounders included session effects modeled using linear polynomials, subject estimates of head-motion parameters with 1st order-derivatives (n_parameters_ = 12), motion-contaminated outlier volumes (n_maxframes_ = 64), as well as noise signal components from principal component analysis of white matter (n_components_ = 5) and cerebrospinal fluid (n_components_ = 5) compartment signal. After denoising, two quantitative metrics describing brain network organization and capacity for neural adaptation were derived from the processed fMRI data for reliability analysis. The two metrics are described below in 2.4.2. and 2.4.3.

#### 2.4.2 Functional connectivity

Fisher-transformed Pearson correlation coefficients denoting the functional connectivity between two brain areas were calculated using the mean fMRI time series of brain atlas parcels computed and stored by CONN (conn*/results/preprocessing/ROI_Subject*_Condition*.mat). For each subject and fMRI run, a whole brain connectivity matrix was constructed plus submatrices for the motor, limbic, and cognitive networks impacted by DBS in PD (Fig. 1D, **Suppl. Table 1**).

#### 2.4.3 Brain variability

Often computed as the standard deviation of voxel-wise fMRI time series averaged within a single brain atlas parcel (Fig. 1D), here brain variability was calculated for each subject, fMRI run, and brain atlas parcel and stored as separate vectors for the whole brain, motor, limbic, and cognitive networks. Mean fMRI time series corresponding to each brain atlas parcel were extracted as described in 2.4.2.

### 2.5 Reliability analysis

#### 2.5.1 Effects of stimulation and interval time

For each participant (n = 16), stimulation condition (n = 2), and brain network (n = 4), the reliability of test and retest connectivity matrices and brain variability metrics was calculated as single-subject intra-class correlation coefficients (ICC). We calculated the absolute agreement of repeated measures using a two-way model (A-1 model, MATLAB File Exchange: 22099-intraclass-correlation-coefficient-icc) (McGraw and Wong, 1996) that accepts a subject-specific *N x M* matrix of vectorized test and retest fMRI metrics with *N* equal to the total number of parcels or connections in a network and *M* equal to 2: the test and retest condition. To use ICC in this way, we assumed that the set of brain regions could be approximately considered a random sample. After calculating ICC, Wilcoxon signed-rank tests, and Pearson correlations evaluated network-specific agreement of ICC values across on and off stimulation conditions at the group level. The results were generated twice using fMRI data processed at a standard 0.1 Hz and an exploratory 0.25 Hz lowpass filter (see 2.4.1.), the latter allowing us to assess the reliability of higher-frequency signals that may contain true neuronal activity but also increased physiological noise. For this initial analysis and all analyses described below, potential statistical significance was determine based on a nominal p-value of p < 0.5. Given our small sample size and the predominately exploratory nature of the work, we did not apply formal adjustments for multiple testing, but instead relied on scientific judgement of effect sizes and confidence intervals to assess where caution is warranted despite findings with p < 0.05.

In the subset of three patients who returned for repeat test–retest imaging, the effects of stimulation were further explored across shorter (<1 min) versus longer (2.3–6.4 months) test–retest interval times (see **Suppl. Fig. 3A**). Using the same methods as above, ICC values were computed for all combinations of test and retest fMRI metrics within and across the first and second imaging time points. Exploratory Wilcoxon tests and Pearson correlations contrasted ICC values across stimulation conditions and test–retest interval times. To increase our sample for these exploratory statistical comparisons, subjects’ multiple ICC values for different brain networks and between-session test–retest permutations were evaluated independently.

#### 2.5.2 Effects of DBS metal artifact

To evaluate how the DBS metal artifacts impact the reliability of functional connectivity and brain variability metrics, we segmented the brain-hardware related signal loss on each subject’s mean unsmoothed and spatially normalized fMRI images (to ensure accurate visualization and labeling of affected brain voxels). Segmentation was achieved using a fast and automated intensity-based Otsu thresholding approach in SimpleITK, (Otsu, 1979) followed by refinement of the segmented masks through union with an MNI152 brain mask and manual cleaning in ITK-SNAP (Yushkevich et al., 2006). Binary masks outlining subject-specific metal artifacts were multiplied by the parcellated brain atlas (Zhang et al., 2018) to identify all parcels containing metal artifacts (see Fig. 4A). Subjects’ ICC values for each stimulation condition were extracted for fMRI metrics restricted to affected versus unaffected atlas parcels, and Wilcoxon rank-sum tests were employed to test for an effect of metal artifact on fMRI metric reliability.

#### 2.5.3 Sources of between-subject variance in reliability

We used a hypothesis-based approach to investigate factors that might explain subject differences in test–retest reliability for each stimulation condition. For the DBS-OFF condition, we hypothesized that residual head motion during scanning and, relatedly, the severity of motor symptoms that produce or slow movement would best explain subject differences in data reliability. To test this, Pearson correlations compared DBS-OFF ICCs with the difference in 95th percentile framewise displacement values for the test and retest scans and raw motor sub-scores for tremor, rigidity, and bradykinesia symptoms. For the DBS-ON condition, we expected reliability to predominantly reflect the direct time-varying effects of stimulation on the brain, as well as the corresponding symptom response to DBS and its presumed accompanying reduction in propensity to move during scanning. Thus, separately, DBS-ON ICCs were correlated with acute functional brain response to DBS (computed as the mean difference in whole-brain connectivity across DBS-ON and DBS-OFF conditions) and acute MDS-UPDRS-III improvement with DBS. Finally, for all brain networks, Pearson correlations and Wilcoxon rank-sum tests were used to explore the potential added effects of DBS target and age on reliability.

## 3 Results

### 3.1 Effects of stimulation and interval time

**Fig. 2:**
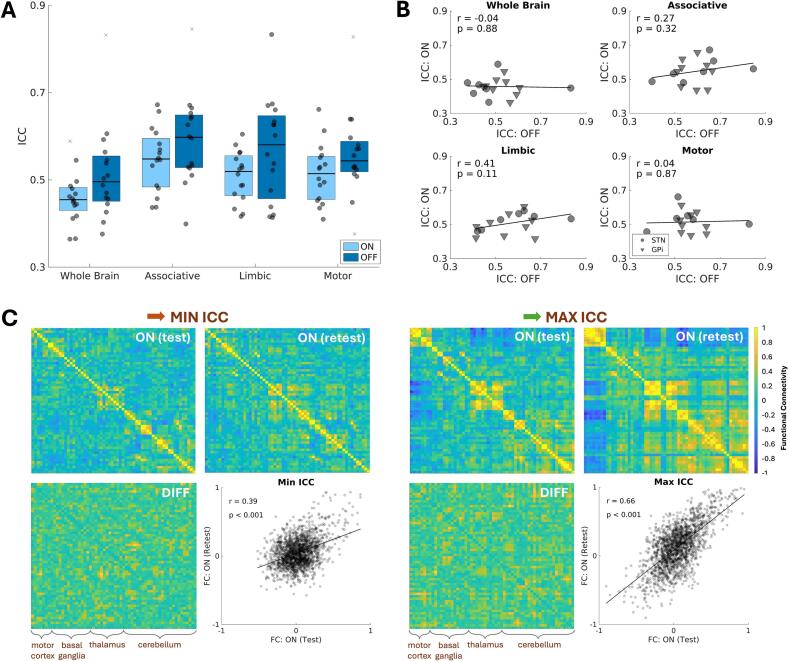
Effects of brain stimulation on functional connectivity reproducibility. A-B. Within-session test–retest reproducibility—measured via the intraclass correlation coefficient (ICC)—of whole-brain and network-specific functional connectivity (FC) did not change significantly when DBS was turned off versus on. C. Example test and retest connectivity matrices and vectorized scatterplots for the two patients with the minimum (red arrow) and maximum (green arrow) ICC when DBS was turned on. DIFF = absolute value difference. (For interpretation of the references to colour in this figure legend, the reader is referred to the web version of this article.)

Uncorrected p-values, effect sizes, and confidence intervals for all comparisons are summarized in Table 2. Across subjects, networks, and stimulation conditions, within-session ICCs ranged from 0.36 to 0.85 for brain connectivity metrics (Fig. 2A). On average, ICCs were lowest for the whole-brain network (0.49±0.09), followed by the motor (0.54±0.09), limbic (0.54±0.10), then associative network (0.59±0.10). Within-session reliability of connectivity metrics was not significantly (p_uncorrected_ < 0.05) different across stimulation conditions; however, on average, metrics trended as more reproducible when DBS was turned off (Fig. 2A). Plotting DBS-ON ICCs against DBS-OFF ICCs revealed weak yet plausible linear relationships for non-motor networks (Fig. 2B). Fig. 2C shows test–retest connectivity matrices for patients with the highest and lowest reliability, with detailed atlas labels in **Suppl. Fig. 1**.

**Table 2:** Summary of main statistical findings.

	Statistical significance		
Effect	p-value	effect size^a^	95% CI
**Effect of stimulation,**Fig. 2A *(connectivity metric, signed-rank test)* whole-brain motor limbic associative	0.16 0.15 0.09 0.13	*δ* = 0.38 *δ* = 0.30 *δ* = 0.32 *δ* = 0.24	[-8.33e-03, 0.73] [-0.12, 0.66] [-0.04, 0.65] [-0.06, 0.58]
**Effect of stimulation,**Fig. 2B *(connectivity metric, correlation test)* whole-brain motor limbic associative	0.88 0.87 0.11 0.32	r = -0.04 r = 0.04 r = 0.41 r = 0.27	[-0.52, 0.46] [-0.46, 0.53] [-0.11, 0.75] [-0.26, 0.67]
**Effect of stimulation,**Fig. 3A *(variability metric, signed-rank test)* whole-brain motor limbic associative	0.20 0.16 0.50 0.57	*δ* = 0.10 *δ* = 0.14 *δ* = 0.09 *δ* = 0.02	[-0.23, 0.36] [-0.17, 0.43] [-0.27, 0.36] [-0.29, 0.25]
**Effect of stimulation,**Fig. 3B *(variability metric, correlation test)* whole-brain motor limbic associative	0.01 <0.001 0.06 <0.01	r = 0.61 r = 0.76 r = 0.48 r = 0.65	[0.17, 0.85] [0.42, 0.91] [-0.03, 0.79] [0.22, 0.87]
**Effects of metal artifact,**Fig. 4C *(connectivity metric, signed-rank test)* DBS-ON, affected vs. unaffected DBS-ON, affected vs. whole-brain DBS-ON, unaffected vs. whole-brain DBS-OFF, affected vs. unaffected DBS-OFF, affected vs. whole-brain DBS-OFF, unaffected vs. whole-brain	<0.001 <0.001 <0.001 <0.01 <0.01 <0.01	*δ* = 0.50 *δ* = 0.38 δ = -0.18 *δ* = 0.48 *δ* = 0.37 δ = -0.08	[0.23, 0.74] [0.15, 0.60] [-0.34, −0.03] [0.23, 0.71] [0.15, 0.58] [-0.16, 0.08]
**Effects of metal artifact,**Fig. 4C *(variability metric, signed-rank test)* DBS-ON, affected vs. unaffected DBS-ON, affected vs. whole-brain DBS-ON, unaffected vs. whole-brain DBS-OFF, affected vs. unaffected DBS-OFF, affected vs. whole-brain DBS-OFF, unaffected vs. whole-brain	0.02 0.06 <0.01 0.02 0.04 <0.01	δ = -0.41 δ = -0.16 *δ* = 0.29 δ = -0.22 *δ* = 0.22 δ = -0.07	[-0.73, −0.03] [-0.42, 0.15] [0.06, 0.55] [-0.45, 0.06] [-0.18, 0.21] [0.04, 0.45]
**Effects of other factors,**Fig. 5**A–C** *(connectivity metric, correlation/rank-sum test)* DBS-OFF, head motion DBS-OFF, tremor DBS-OFF, rigidity + bradykinesia DBS-ON, total motor improvement DBS-ON, total motor improvement x connectivity response DBS-ON, brain target, whole brain DBS-ON, brain target, motor DBS-ON, brain target, limbic DBS-ON, brain target, associative DBS-OFF, brain target, whole brain DBS-OFF, brain target, motor DBS-OFF, brain target, limbic DBS-OFF, brain target, associative	0.19 0.33 0.27 0.99 0.83 0.92 0.76 0.25 0.92 0.14 0.21 0.84 0.68	r = -0.35 r = 0.26 r = 0.29 r = -0.004 r = 0.06 δ = -0.05 *δ* = 0.11 *δ* = 0.37 *δ* = 0.05 δ = -0.46 δ = -0.40 *δ* = 0.08 *δ* = 0.14	[-0.72, 0.18] [-0.27, 0.67] [-0.24, 0.69] [-0.50, 0.49] [-0.45, 0.54] [-0.67, 0.59] [-0.56, 0.69] [-0.34, 0.83] [-0.59, 0.67] [-0.90, 0.34] [-0.90, 0.42] [-0.56, 0.67] [-0.60, 0.77]
**Effects of other factors,**Fig. 5**A–C** *(variability metric, correlation/rank-sum test)* DBS-OFF, head motion DBS-OFF, tremor DBS-OFF, rigidity + bradykinesia DBS-ON, total motor improvement DBS-ON, total motor improvement x connectivity response DBS-ON, brain target, whole brain DBS-ON, brain target, motor DBS-ON, brain target, limbic DBS-ON, brain target, associative DBS-OFF, brain target, whole brain DBS-OFF, brain target, motor DBS-OFF, brain target, limbic DBS-OFF, brain target, associative	0.02 0.52 0.64 0.06 0.11 0.07 <0.01 0.02 0.02 0.02 0.07 <0.01 <0.01	r = -0.58 r = 0.17 r = -0.13 r = 0.47 r = 0.42 *δ* = 0.56 *δ* = 0.81 *δ* = 0.71 *δ* = 0.71 *δ* = 0.68 *δ* = 0.56 *δ* = 0.78 *δ* = 0.84	[-0.84, −0.12] [-0.35, 0.62] [-0.59, 0.39] [-0.03, 0.79] [-0.10, 0.76] [-0.13, 0.90] [0.00, 1.00] [0.07, 1.00] [0.08, 1.00] [-0.23, 1.00] [-0.16, 0.93] [-0.17, 1.00] [0.18, 1.00]

a effect sizes were computed as Pearson’s coefficient, r, and Cliff’s delta, δ, for correlation tests and Wilcoxon tests, respectively.

**Fig. 3:**
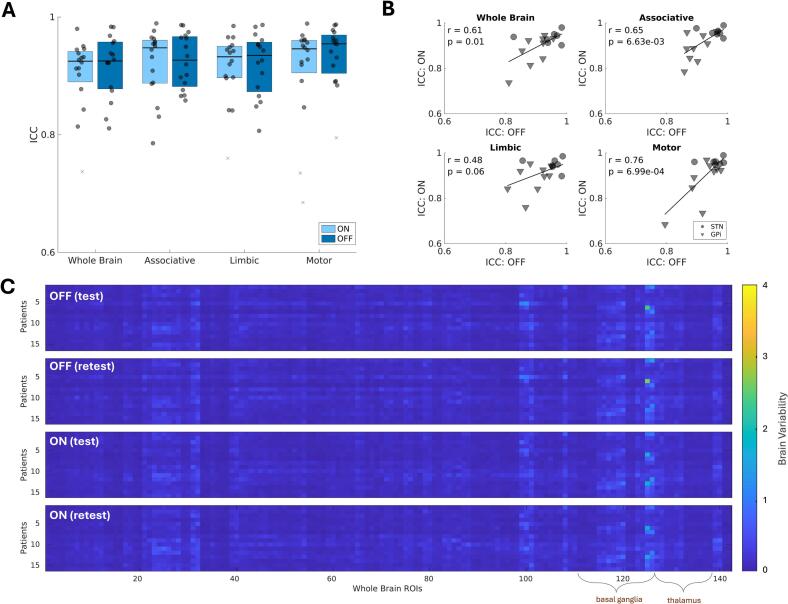
Effects of brain stimulation on brain variability reproducibility. A-B. Test-retest reproducibility—measured via the intraclass correlation coefficient (ICC)—of whole-brain and network-specific brain variability did not change significantly when deep brain stimulation (DBS) was turned off versus on. ICC values were higher and more clustered for subthalamus nucleus (STN)- compared to globus pallidus internus (GPi)-treated patients. C. Heat map of the 16 patients (rows) brain variability values measured within each whole-brain region-of-interest (ROI; columns) for the test and retest, DBS ON and OFF conditions.

Of the two metrics examined, compared to brain connectivity metrics, within-session ICCs for brain variability metrics were numerically higher (range across subjects, networks, stimulation conditions: 0.68–0.99; Fig. 3A). There were no detected differences in ICC across stimulation conditions (Fig. 3A). This was also reflected in the subject-level plots where DBS-ON ICCs and DBS-OFF ICCs were significantly (p_uncorrected_ < 0.05) correlated for all networks but the limbic network, notably, with numerically higher and more visually clustered data points for STN- compared to GPi-treated patients (Fig. 3B; r_wholebrain_ = 0.61, 95%CI:[0.17,0.85]; r_associative_ = 0.65, 95%CI: [0.22,0.87]; r_motor_ = 0.76, 95%CI:[0.42, 0.91]). (Fig. 3C shows test–retest brain variability heat maps for all subjects, with detailed atlas labels in **Suppl. Fig. 1**.) These main effects of brain stimulation on the reliability of both fMRI metrics could be reproduced using data filtered with a wider bandpass filter of 0.01–0.25 Hz (**Suppl. Fig. 2**).

In the three subjects who underwent repeat imaging 2.3–6.4 months later, the within-session test–retest reliability of DBS-OFF connectivity measures appeared consistent across serial visits (**Suppl. Fig. 3A-B**; r = 0.60, 95%CI:[0.04, 0.87]). Within-session reliability also appeared to be higher than between-session reliability for both stimulation conditions (**Suppl. Fig. 3B**; δ_DBS-ON_ = 0.42, 95%CI:[0.13,0.64]; δ_DBS-OFF_ = 0.39, 95%CI:[0.13,0.61]). Similar patterns were observed for brain variability (**Suppl. Fig. 3C**; δ_DBS-ON_ = 0.59, 95%CI:[0.35,0.77]; δ_DBS-OFF_ = 0.34, 95%CI:[0.04,0.58]), with corresponding ICCs numerically higher and less variable across patients and networks than those observed for connectivity.

### 3.2 Effects of DBS metal artifact

**Fig. 4:**
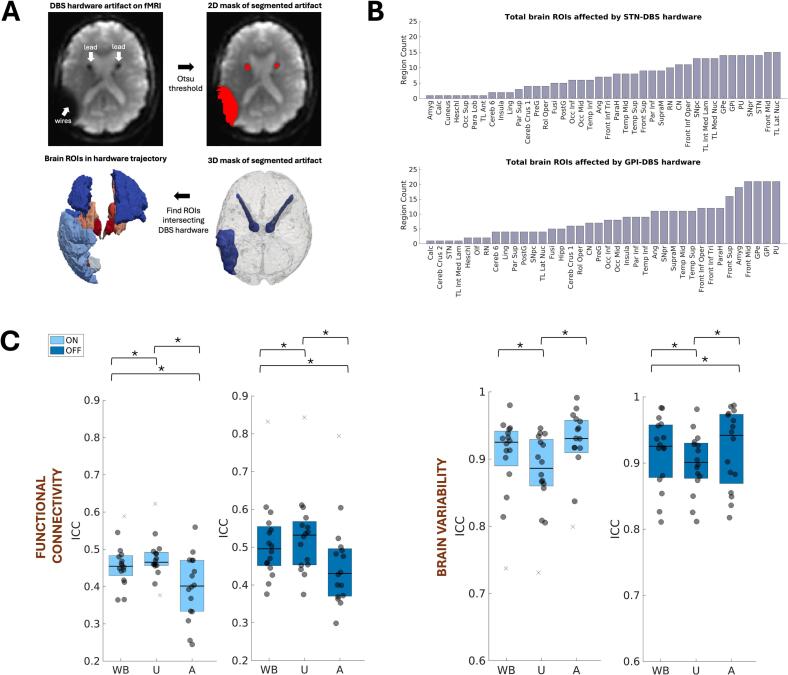
Effects of DBS metal artifact. A-B. Metal artifacts on fMRI images were segmented, and affected brain regions of interest (ROIs) intersecting the artifacts were identified. C. Affected connectivity metrics were less reproducible than unaffected ones, while the opposite effect was observed for brain variability metrics. WB = whole brain, A = affected, U = unaffected. *p < 0.05.

Segmentation of the metal artifacts revealed multiple areas affected by signal loss, including the basal ganglia, thalamus, and some frontal, parietal, temporal, and occipital regions (Fig. 4**A–B**). Affected areas showed significantly (p_uncorrected_ < 0.05) lower reproducibility in brain connectivity metrics (Fig. 4C; δ_DBS-ON_ = 0.50, 95%CI:[0.23,0.74]; δ_DBS-OFF_ = 0.48, 95%CI:[0.23,0.71]), yet significantly (p_uncorrected_ < 0.05) higher reproducibility in brain variability metrics, compared to unaffected areas (Fig. 4C; δ_DBS-ON_ = -0.41, 95%CI:[-0.73,-0.03]; δ_DBS-OFF_ = -0.22, 95%CI:[-0.45,0.06]).

### 3.3 Sources of between-subject variance in reliability

**Fig. 5:**
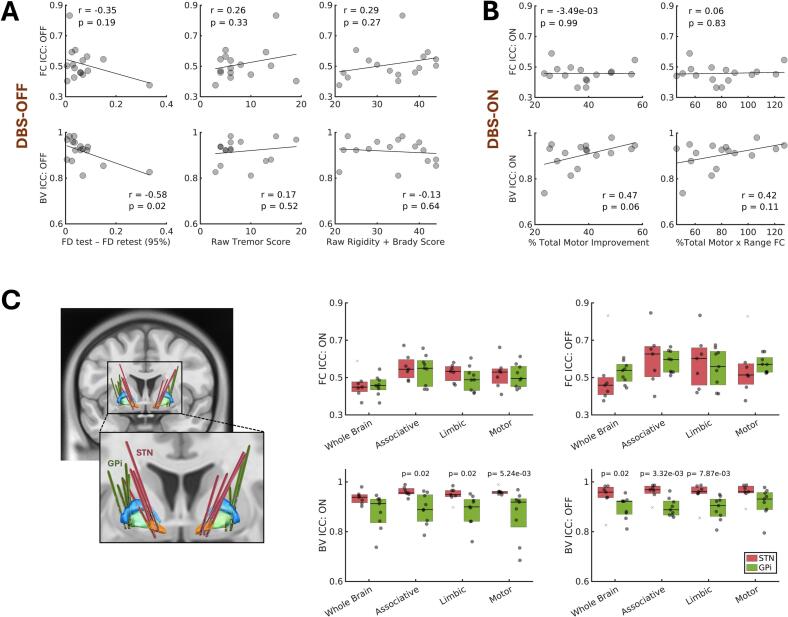
Sources of between-subject variance in ICC. A–B. Framewise displacement (FD) during fMRI, as well as clinical and functional connectivity (FC) response to DBS—denoted as *% Total Motor* and *Range of FC*— could explain some between-subject variance in the reproducibility of brain variability (BV) metrics. C. STN-treated patients had more reproducible BV metrics than GPi-treated patients. ICC = intraclass correlation coefficient.

Head motion during fMRI without stimulation significantly (p_uncorrected_ < 0.05) correlated with subject ICCs describing the reliability of whole-brain network variability (Fig. 5A; r = −0.58, 95%CI:[−0.84,-0.12]). A similar pattern was observed for connectivity, while clinical motor scores showed weaker effects and varying patterns that did not consistently align with our hypotheses. Although not significant (p_uncorrected_ < 0.05), with DBS active during fMRI, patients’ clinical and functional brain response to DBS appeared to better explain between-subject differences in the reliability of brain variability than connectivity metrics (Fig. 5B). Of the other factors explored, there were no detected effects of age, though patients with STN implants had significantly (p_uncorrected_ < 0.05) more reproducible brain variability metrics than those with GPi implants across stimulation conditions and most brain networks (Fig. 5C; δ_DBS-ON_motor_ = 0.81, 95%CI:[0.00,1.00]; δ_DBS-ON_limbic_ = 0.71, 95%CI:[0.07,1.00]; δ_DBS-ON_associative_ = 0.71, 95%CI:[0.08,1.00]; δ_DBS-OFF_wholebrain_ = 0.68, 95%CI:[-0.23,1.00]; δ_DBS-OFF_limbic_ = 0.78, 95%CI:[-0.17,1.00]; δ_DBS-OFF_associative_ = 0.84, 95%CI:[0.18,1.00]). This effect of brain target was absent for connectivity metrics.

## 4 Discussion

Here, we aimed to explore the reliability of rsfMRI metrics in the presence of implanted DBS leads and during active stimulation. Two prior studies in PD performed subanalyses of same-session test–retest fMRI reliability using block-design STN-DBS paradigms, with stimulation repeatedly cycled ON and OFF. (Hancu et al., 2019, Shen et al., 2020) In 4 patients, Hancu et al. reported good qualitative agreement between activation maps acquired at 1.5T and 3T (Hancu et al., 2019). More recently, in 14 patients, Shen et al. measured ICC values ranging from 0.59 to 0.70 for brain activation maps acquired across different time points and frequency settings (Shen et al., 2020). This study adds to these works by uniquely exploring the reliability of resting-state data acquired during fixed stimulation conditions (where DBS is either turned ON or OFF for the entire fMRI run), as well as across different DBS targets and serial scan sessions, while also exploring possible sources underlying between-subject variations in ICC.

Among the fMRI metrics considered, network-level ICC values were numerically higher for brain variability metrics than for brain connectivity metrics. While the reliability of individual fMRI connections has consistently been demonstrated to be poor in even healthy populations, (Noble et al., 2019) better reliability, as observed here, has been reported for network-level connectivity measures (Elliott et al., 2019, Guo et al., 2012, Zuo and Xing, 2014). Further supporting our results are reports of good reliability for voxel-wise fMRI measures of brain variability and analogous measures of the amplitude of low-frequency fluctuations (ALFF) (Wehrheim et al., 2024, Zhao et al., 2018, Zuo et al., 2010). Indeed, Zuo and colleagues showed that voxel-wise ALFF has high within- and between-session reliability, with over 80% of gray matter voxels producing ICC values greater than 0.85. (Zuo et al., 2010) The seemingly superior reliability of brain variability observed in our analyses—including in networks not directly affected by DBS metal artifact—may be attributed to the metric’s sole reliance on the magnitude of fMRI signals within a single brain parcel, in contrast to connectivity metrics, which require temporally correlated signal structure between two distinct brain parcels. The proximity of each brain parcel to focal stimulation can influence how the corresponding fMRI signal is modulated by DBS (Gollo et al., 2017), essentially introducing more noise into the connectivity correlation. This was reflected in our results, which showed that active stimulation during fMRI (compared to no stimulation) may be associated with lower reliability of connectivity metrics, especially at the whole-brain network level where stimulation effects are highly spatially heterogeneous and vary most strongly across brain connections. Interestingly, altering the stimulation condition did not appear to alter brain variability ICCs, possibly reinforcing prior evidence of the reliability and robustness of this promising yet underutilized metric that has been shown to facilitate brain network organization and integration, as well as predict therapeutic outcomes (Baracchini et al., 2021, Garrett et al., 2018, Månsson et al., 2022, Morrison et al., 2022).

Within-session DBS effects on fMRI reliability were replicable within our exploratory sample of three patients who returned months later and repeated the test–retest scan protocol. In line with prior investigations of test–retest interval times, (Zhao et al., 2018, Birn et al., 2013) within-session reliability appeared better than between-session reliability. Some between-session ICCs were very poor (<0.3), which in PD could reflect day-to-day fluctuations in symptoms that contribute to physiological noise and motion during MRI (Jankovic, 2005, Noack et al., 2014). For now this might underscore the importance of quantifying reliability and carefully interpretating results from DBS-fMRI studies employing multi-session designs (such as in our prior work (Slepneva et al., 2024), and using practices like multi-session data concatenation, (Laumann et al., 2015) in the case of DBS, to overcome manufacturer limits on scan time and/or increase statistical power.

While limits on scan time and other system parameters impact achievable image quality, T2*w fMRI signal loss caused by the DBS implant is often of greatest concern with researchers using various strategies to address the artifacts (Horn et al., 2019, In et al., 2017, Lehto et al., 2017, Mueller et al., 2018, Zhang et al., 2021). Here, we show for the first time that connectivity measures involving brain areas affected by this metal artifact may be less reproducible than those only involving unaffected regions. This is valuable information as the volume of tissue activated by DBS, centered on the lead artifact, is frequently used in fMRI research for biomarker development. Assuming patients’ brain hardware does not move across scan sessions, we hypothesize that this effect of metal artifact could be due to active or residual effects of stimulation, along with partial volume effects and more substantial differences in fMRI signal for connections involving one affected and one unaffected brain parcel. As for brain variability metrics, which were more reproducible on average in parcels affected by metal artifacts, this opposing effect could be due to T2*w signal loss attenuating temporal signal variability, resulting in artificially inflated stability.

Lastly, we aimed to investigate sources that might be influencing between-subject ICC variation. With DBS turned off and symptoms reemerging, greater differences in head motion across test and retest runs seemed to lower metric reliability. Clinical scores like tremor severity did not echo this trend, perhaps because our tremor sub-scores reflect the combined severity of rest and action tremor assessed in an environment different from resting conditions in the scanner. When DBS was turned on, individuals with greater total clinical response and functional brain response to DBS appeared to have more reliable brain variability metrics. This could be an effect of symptom improvement leading to less motion during scanning; however, it could also reflect a direct stimulation effect on the fMRI signal. While we hypothesized that time-varying DBS effects on brain activity would diminish and not improve fMRI reliability, connectivity measures did show subtle trends that may still support this hypothesis. With a larger sample and the ability to model multiple covariates, we can approach a better understanding of the relative impact of these effects on fMRI reliability. This includes the effects of treatment factors like the chosen brain target, as we found that STN-treated patients had more reliable brain variability metrics across all networks. Compared to the GPi, an output nucleus of the basal ganglia, the STN is a smaller relay structure with clearly delineated functional subregions that project to motor, limbic, and associative areas (Prasad and Wallén-Mackenzie, 2024, Williams et al., 2014). Consistent modulation of these functionally distinct STN subareas might contribute to more synchronous brain activity and thus enhanced fMRI reliability, as could related factors like lead positioning which dictates the degree of network engagement (Horn et al., 2019, Basich-Pease et al., 2024). Alternatively, this findings could reflect underlying differences in individual brain network integrity, since patients receiving GPi DBS are often at greater risk for cognitive decline (Okun et al., 2009) and may therefore have more brain atrophy impacting network function.

Looking forward, this early evidence for DBS altering fMRI reliability highlights a need for standardized reporting of reliability to aid interpretation of results, including the validity of new findings and the (in-)ability to reproduce previously reported biomarkers. Through such standardization, we can identify both reliable and reproducible biomarkers for clinical DBS applications. While our study represents a critical step toward this goal, several limitations should be noted and considered when interpreting the results. Our sample size was small relative to typical non-DBS fMRI reliability studies, limiting statistical power, formal adjustments for multiple testing, and covariate modeling. ICC calculations for connectivity matrices further assumed that brain parcels could be treated as a random sample and independent, despite known spatial correlations across regions, and stimulation washout times varied across patients. Despite these limitations, our findings address an important gap and establish a foundation for future studies to more rigorously assess fMRI reliability in the context of DBS, including across other DBS indications.

## 5 Conclusion

Here, we demonstrate that the presence of DBS hardware and active stimulation may lower the reliability of rsfMRI metrics. To develop clinically useful fMRI biomarkers for DBS and aid assessments of biomarker reproducibly across studies, the reliability of single study results need to be reported.

## 6 Disclosures

P.A.S. receives support from Medtronic and Boston Scientific for fellowship education. J.L.O. receives support from Medtronic and Boston Scientific for research and education and consults for AbbVie. D.D.W. receives support from Boston Scientific, and consults for Boston Scientific, Medtronic, and Iota Biosciences. S.D., J.M., L.R., A.F., S.W., J.K., I.O.B. and M.A.M. declare no competing interests.

## Ethics Statement

This study was approved by the ethics committee of the University of California, San Francisco. Written informed consent was obtain from all study participants prior to participation and all prospective procedures performed were conducted in accordance with the ethical standards of the institutional research committee.

## CRediT authorship contribution statement

**Skyler Deutsch:** Writing – review & editing, Writing – original draft, Project administration, Methodology, Formal analysis, Data curation. **Juhi Mehta:** Writing – review & editing, Writing – original draft, Methodology, Formal analysis, Data curation. **Lee B. Reid:** Writing – review & editing, Methodology. **Andrea Fuentes:** Writing – review & editing, Data curation. **Sarah Wang:** Writing – review & editing, Resources, Project administration. **John Kornak:** Writing – review & editing, Methodology. **Philip A. Starr:** Writing – review & editing. **Jill L. Ostrem:** Writing – review & editing, Resources. **Doris D. Wang:** Writing – review & editing. **Ian O. Bledsoe:** Writing – review & editing, Resources, Data curation. **Melanie A. Morrison:** Writing – review & editing, Writing – original draft, Supervision, Resources, Project administration, Methodology, Funding acquisition, Formal analysis, Data curation, Conceptualization.

## Declaration of competing interest

The authors declare that they have no known competing financial interests or personal relationships that could have appeared to influence the work reported in this paper.

## Data Availability

Data and code are available on GitHub and OpenNeuro via links in the manuscript.
