# Supplementary material for: Test-retest reliability of resting-state functional magnetic resonance imaging during deep brain stimulation for Parkinson’s disease
Source: Neuroimage Clin. 2026 Feb 18;49:103973. doi: 10.1016/j.nicl.2026.103973 (PMC12955080; doi:10.1016/j.nicl.2026.103973)
Supplement: Supplementary Data 1 [file mmc1.pdf]

Supplementary Materials

Test-retest reliability of resting-state functional magnetic resonance imaging during deep brain stimulation for Parkinson's disease

Skyler Deutsch BS[1a], Juhi Mehta BS[1a], Lee B. Reid PhD[1], Andrea Fuentes MD[2], Sarah Wang PhD[3], John Kornak PhD[4], Philip A. Starr MD PhD[5], Jill Ostrem MD[3], Doris D. Wang MD PhD[5], Ian O. Bledsoe MD[3], Melanie A. Morrison PhD[1]

[a] Skyler Deutsch and Juhi Mehta are joint co-first authors

[1] Department of Radiology & Biomedical Imaging, University of California San Francisco, USA

[2] Department of Neurology & Neurological Sciences, Stanford University, Stanford, CA, USA

[3] Department of Neurology, University of California San Francisco, San Francisco, CA, USA

[4] Department of Epidemiology and Biostatistics, University of California San Francisco, CA, USA

[5] Department of Neurological Surgery, University of California San Francisco, San Francisco, CA, USA

(1) Summary of fMRI preprocessing steps:

Functional and T1w data were preprocessed using the default preprocessing pipeline for volume-based analyses in CONN (v22a),[1] an open-source SPM-based (v12)[2] toolbox that can run stand-alone and in MATLAB (MathWorks, Natick, MA). The only addition to this pipeline was the initial removal of two non-steady-state fMRI volumes. Otherwise, the steps were as follows: (1) functional realignment and resampling of fMRI volumes to the first steady-state volume of the session using b-spline interpolation, (2) temporal alignment and resampling of fMRI data using sinc-interpolation and interleaved slice acquisition times from the BIDS .json files, (3) identification of outlier fMRI volumes for denoising with a framewise displacement or global signal change greater than 0.9mm or 5sd, respectively, (4) probabilistic tissue classification for denoising via segmentation of tissue compartments from fMRI and T1w reference images, (5) normalizing and resampling the data to standard Montreal Neurological Institute (MNI) space via nonlinear spatial transformations, and (6) spatial smoothing of the normalized fMRI data using the default 8mm Gaussian kernel to enhance data signal-to-noise ratio (SNR) for subsequent network analysis. The process of normalizing the data enabled the use of brain atlases to extract region-specific time series

and calculate the fMRI metrics described below. For this study, we manually imported the Zhang atlas[3] into CONN, derived from the standard automated anatomical labeling atlas[4] and iron-sensitive imaging data for superior segmentation of deep grey matter structures.

(2) Calculating MDS-UPDRS-III symptom subscores:

Raw MDS-UPDRS-III symptom subscores were also used as a proxy of disease severity in our variance analysis (see 2.5.3.) and calculated as in Li et al.[5] using the following exam items: bradykinesia (Items 3.2, 3.4-3.9, 3.14), rigidity (Item 3.3), tremor (Items 3.15-3.18), and axial symptoms (Items 3.1, 3.9-3.13). A template that calculates these scores is available on our GitHub repository.

Supplementary Table 1. Brain networks of interest.

	Network			
Brain Region	Whole Brain	*Motor	*Limbic	*Associative
Precentral	X	X		
Frontal-Sup-2/Mid-2	X			X
Frontal-Inf-Oper/Tri/Orb	X			X
Rolandic-Oper	X			X
Supp-Motor-Area	X	X		
Olfactory	X			
Frontal-Sup-Medial	X			X
Frontal-Med-Orb	X			X
Rectus	X			
OFC-Med/Ant/Post/Lat	X		X	
Insula	X		X	
Cingulate-Ant	X		X	
Cingulate-Mid/Post	X			
Parahippocampal	X		X	
Calcarine	X			
Cuneus	X			
Lingual	X			
Occipital-Sup/Mid/Inf	X			
Fusiform	X			
Postcentral	X	X		
Parietal-Sup/Inf	X			
Supramarginal	X			X
Angular	X			X
Precuneus	X			
Paracentral-Lobule	X	X		

Heschl	X			
Temporal-Sup	X			
Temporal-Pole-Sup	X		X	
Temporal-Mid	X			
Temporal-Pole-Mid	X		X	
Temporal-Inf	X			
Cerebelum-Crus1/Crus2	X	X		
Cerebelum-3 to 10	X	X		
Vermis-1/2 to 10	X	X		
Caudate-Nucleus	X	X	X	X
Putamen	X	X	X	X
Substantia-Nigra-PR/PC	X	X	X	X
Red-Nucleus	X			
Dentate-Nucleus	X			
TL-Anterior	X	X	X	X
TL-Medial-Nucleus	X	X	X	X
TL-Midline-Thalamic-Nuclei	X	X	X	X
TL-Pulvinar	X	X	X	X
TL-Internal-Medullary-Lamina	X	X	X	X
TL-Lateral-Nucleus	X	X	X	X
Amygdala	X		X	
Hippocampus	X		X	
Globus-Pallidus-I/E	X	X	X	X
Subthalamic-Nucleus	X	X	X	X

*****Brain atlas parcels from the Zhang atlas[3] were assigned to each subnetwork based on a broad review of prior literature on functional brain organization.

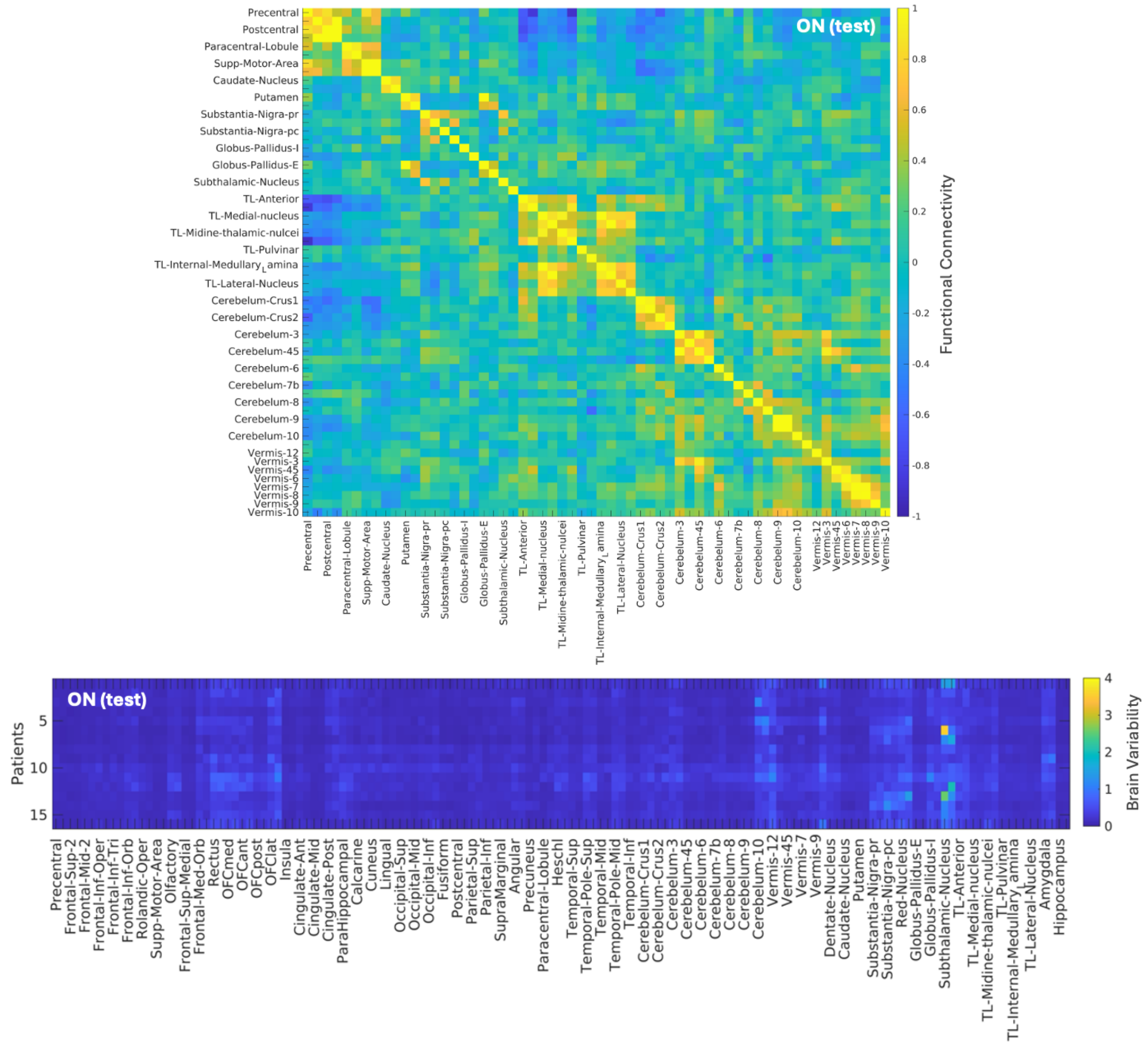

Supplementary Figure 1. Atlas labels for connectivity matrices and brain variability heat maps shown in main Figures 1 and 2.

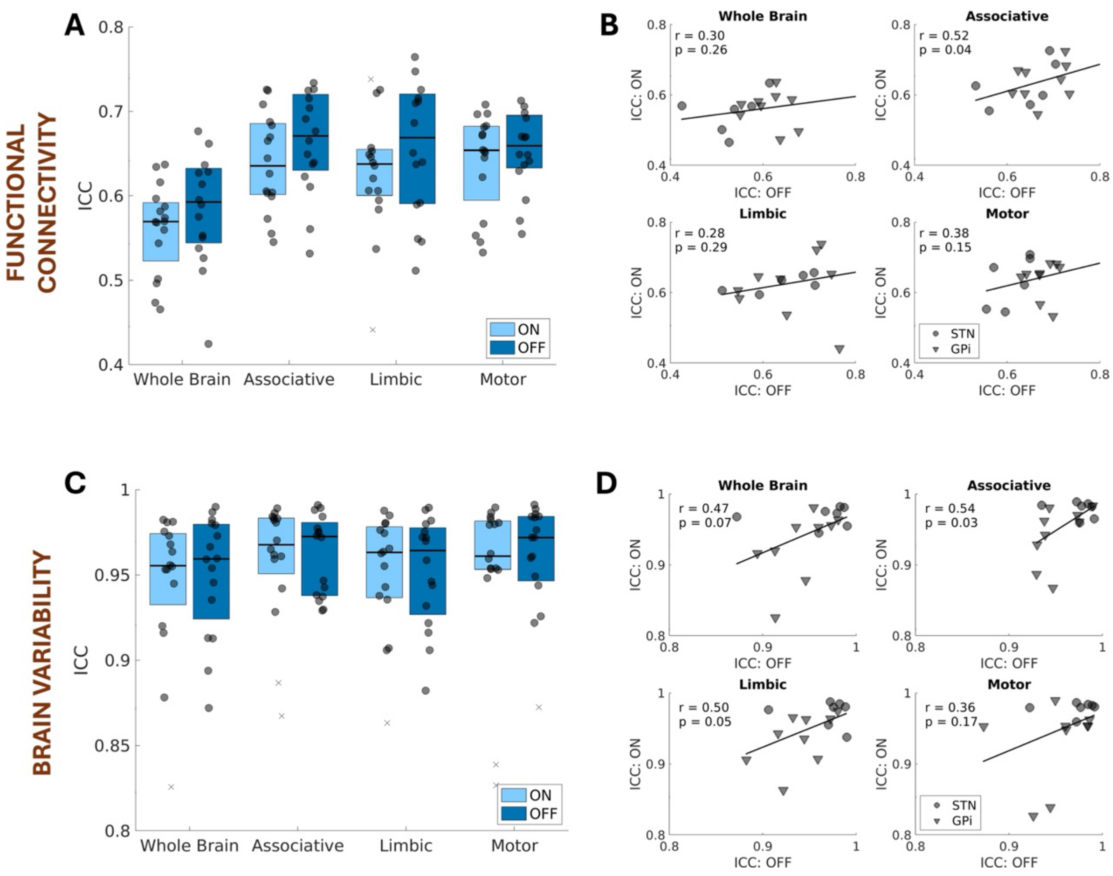

Supplementary Figure 2. Effects of brain stimulation on reproducibility of fMRI data denoised with a wider bandpass filter. Test-retest reproducibility—measured via the intraclass correlation coefficient (ICC)—of whole-brain and network-specific functional connectivity (**A-B**) and brain variability (**C-D**) metrics, derived from fMRI data bandpass filtered at 0.01-0.25 Hz. Note: ICC scales differ across the two functional metrics to aid visualization of trends in the data across stimulation conditions.

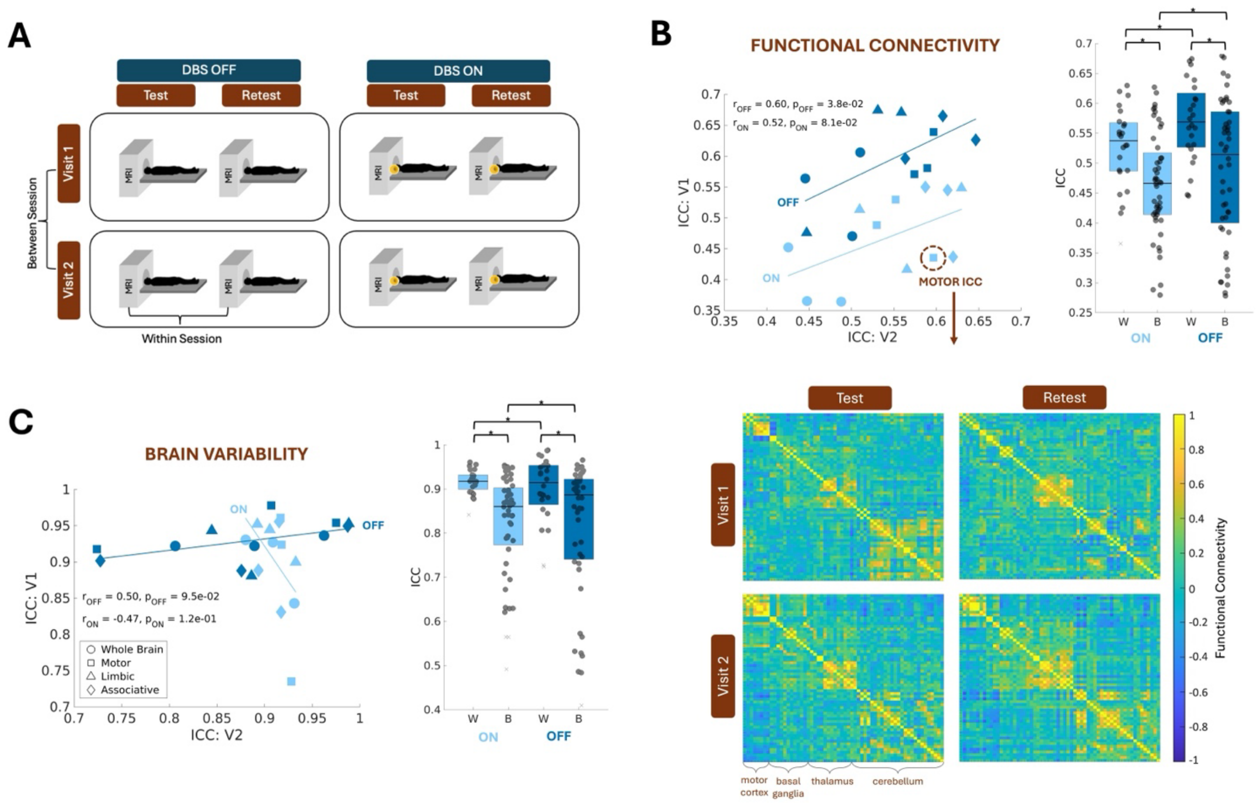

Supplementary Figure 3. Effects of test-retest interval time. A. Test and retest fMRI data were acquired twice during two separate scanning sessions (V1 & V2). The data were used to evaluate fMRI reproducibility within (W) and between (B) sessions and across stimulation conditions. **B.** Within-session reproducibility of whole-brain and network-specific functional connectivity matrices—measured via the intraclass correlation coefficient (ICC)—slightly differed across scanning sessions but appeared better than between-session ICC values. This is illustrated for the motor network in one representative patient example. **C.** Similar effects of interval time were observed for brain variability metrics, which yielded higher ICC values than connectivity metrics across all conditions. Note: ICC scales differ across the two functional metrics to aid visualization of trends in the data across conditions.

References

1. Whitfield-Gabrieli S, Nieto-Castanon A. *Conn* : A Functional Connectivity Toolbox for Correlated and Anticorrelated Brain Networks. *Brain Connect* 2012;2:125–41.

2. Friston KJ, Holmes AP, Worsley KJ, et al. Statistical parametric maps in functional imaging: A general linear approach. *Hum Brain Mapp* 1994;2:189–210.

3. Zhang Y, Wei H, Cronin MJ, et al. Longitudinal atlas for normative human brain development and aging over the lifespan using quantitative susceptibility mapping. *NeuroImage* 2018;171:176–89.

4. Rolls ET, Huang C-C, Lin C-P, et al. Automated anatomical labelling atlas 3. *NeuroImage* 2020;206:116189.

5. Li X, Xing Y, Martin-Bastida A, et al. Patterns of grey matter loss associated with motor subscores in early Parkinson's disease. *NeuroImage Clin* 2018;17:498–504.
